# Alpha-ketoglutarate supplementation ameliorates castor oil-induced diarrhea in mice: *in silico* and *in vivo* insights

**DOI:** 10.1590/1414-431X2026e15456

**Published:** 2026-08-21

**Authors:** A.K.S. Araujo, A.C.P. Oliveira, E.C. Machado, F.B.M. Sousa, K.C. Silva, G. Pacheco, A.L.F. Lopes, L.S. Chaves, A.P. Oliveira, P.S.A. Sousa, J.A. Rocha, M.B. Gois, S.O.T. Klein, L.A.D. Nicolau, J.V.R. Medeiros

**Affiliations:** 1 Laboratório de Inflamação e Gastroenterologia Translacional, Programa de Pós-Graduação em Biotecnologia, Universidade Federal do Delta do Parnaíba, Parnaíba, PI, Brasil; 2 Grupo de Pesquisa em Química Medicinal e Biotecnologia, Universidade Federal do Maranhão, São Bernardo, MA, Brasil; 3 Programa de Pós-Graduação em Biociências e Saúde, Universidade Federal de Rondonópolis, Rondonópolis, MT, Brasil; 4 Centro de Ciências da Saúde, Universidade Federal do Recôncavo da Bahia, Santo Antônio de Jesus, BA, Brasil

**Keywords:** Alpha-ketoglutarate, Castor oil, Diarrhea, Molecular docking, Alpha-ketoglutarate supplementation

## Abstract

Alpha-ketoglutarate (α-kg) is an intermediate of the Krebs cycle and a precursor of glutamine and glutamate. Furthermore, α-kg contributes to the maintenance of intestinal barrier integrity. Diarrhea, a global health problem, is characterized by an increased frequency and liquidity of bowel movements and is closely associated with impaired intestinal function. As oral rehydration is the primary treatment modality available, the present study evaluated the antidiarrheal potential of α-kg in castor oil (CO)-induced diarrhea model and investigated its molecular interactions through *in silico* modeling. Molecular docking was performed to assess the binding affinity of α-kg with CFTR (3SI7), CaCC (5OYB), and Na^+^/K^+^-ATPase (3B8E). For the *in vivo* experiments, mice were divided into the following groups: vehicle, α-kg (120, 60, and 30 mg/kg), CO (10 mL/kg), and loperamide (5 mg/kg). α-kg was administered orally for five consecutive days. On the fifth day, one hour after the final dose, diarrhea was induced with CO. The loperamide group was pre-treated with loperamide prior to CO administration, whereas the CO group received only CO, and the vehicle group received distilled water. After four hours, the animals were euthanized, and the intestinal samples were collected to evaluate intestinal content, chloride ion levels, and oxidative stress markers (malondialdehyde, glutathione, and nitrite). α-kg reduced fecal outputs, intestinal fluid secretion, and oxidative stress markers, while enhancing Na^+^/K^+^-ATPase activity, preserving glutathione levels, and improving histomorphometric parameters and mucin content. In conclusion, α-kg exhibited favorable binding interactions with key molecular, antidiarrheal, and antioxidant effects, and restored intestinal histological parameters.

## Introduction

Alpha-ketoglutarate (α-kg) is a key intermediate in the Krebs cycle, essential for energy metabolism, and a precursor to amino acids, including glutamate and glutamine, which are critical for cellular function, metabolism, and immune function (1,2). Due to this central metabolic role, α-kg and its derivatives have been incorporated into dietary supplementation strategies, either alone or in combination with other amino acids, to increase nutrient availability and support physiological homeostasis (2,3).

Among the amino acids derived from α-kg, glutamine stands out due to its well-documented biological functions in the literature. Scientific evidence indicates that glutamine plays a central role in intestinal integrity, immune regulation, and energy metabolism. It is important to emphasize that glutamine and α-kg are metabolically interconnected, as α-kg serves as a precursor that supports its synthesis, whereas glutamine performs essential functions in muscle and intestinal tissues (3,4). This relationship highlights α-kg as a promising candidate for investigation, as its availability may influence pathways involved in the regulation and recovery of gastrointestinal disorders. This is consistent with its multifaceted biological activities, including the maintenance of intestinal barrier integrity, modulation of the immune response, improvement of metabolism, as well as antioxidant and antineoplastic properties (5,6).

Considering the therapeutic potential of α-kg, it is important to evaluate its possible contribution to gastrointestinal disorders, particularly diarrhea, which is one of the main conditions associated with impaired intestinal integrity. Diarrhea is a significant public health problem, particularly in developing countries, and affects millions of people each year worldwide. It is a gastrointestinal disorder characterized by three or more daily episodes of loose or watery stools that may contain mucus and/or blood depending on the severity (7). Diarrhea is the third leading cause of global morbidity and mortality, with the greatest impact observed in children aged between 1 and 5 years. Its etiology is multifactorial, encompassing viruses, bacteria, parasites, and certain xenobiotics; nevertheless, acute and severe cases are predominantly caused by biological agents, most commonly transmitted through contaminated water or food, underscoring their critical relevance to global health (7,8).

Despite the diversity of etiological factors, pharmacological options for the treatment of diarrhea remain limited. Current management is primarily focused on fluid and electrolyte replacement to prevent dehydration, often complemented by medications aimed at alleviating symptoms associated with increased bowel frequency (9). In this context, the search for effective therapeutic strategies for diarrheal disorders has gained increasing attention in recent years. Emerging evidence suggests that α-kg exerts protective effects on the gastrointestinal tract (10,11). Therefore, the present study aimed to investigate the antidiarrheal effects of α-kg supplementation in a murine model of castor oil-induced diarrhea.

## Material and Methods

### Molecular docking

The three-dimensional structures of proteins potentially involved in the diarrheal process were obtained from the Protein Data Bank (PDB) with the codes: 3SI7 (CFTR), 3B8E (Na^+^/K^+^-ATPase), and 5OYB (CaCC) (11). Docking procedures were performed using the AutoDock 4.2 package (12,13). Proteins and ligands were prepared for molecular docking simulations using AutoDock Tools (ADT) version 1.5.6 (14). The receptor was considered rigid while each ligand was considered flexible. Gasteiger partial charges were calculated after the addition of all hydrogens to the receptors and ligands (15), and non-polar hydrogen atoms were subsequently merged. A cubic grid box measuring 60×60×60 points with a spacing of 0.35 Å between grid points was defined to encompass the entire target. The active sites of selected proteins were identified using the GASS-WEB online server, which is based on genetic algorithms and provides a 99% prediction accuracy (16). The global Lamarckian genetic algorithm (LGA) search and the pseudo-Solis and Wets local search (LS) were the methods applied in molecular docking (17).

### Drugs and reagents

α-kg was purchased from Sigma-Aldrich (USA). All other reagents were obtained from standard commercial suppliers. α-kg and all other drugs were diluted in distilled water prior to use.

### Animals

Mice (Balb/c) of both sexes weighing 25-30 g were obtained from the Federal University of Delta do Parnaíba, Parnaíba, PI, Brazil. The animals were kept under standard laboratory conditions with free access to food and water. This study was submitted to and approved by the Animal Use Ethics Committee of the Federal University of Delta do Parnaíba (CEUA/UFDPar), under protocol number 12/2021.

### Antidiarrheal activity of α-kg in castor oil-induced diarrhea

Castor oil was used to induce diarrhea according to the method described by Awouters et al. (18), with slight modifications. The animals were randomly allocated into six groups, with five animals per group. The groups treated with α-kg were further subdivided based on the dose (30, 60, and 120 mg/kg), with supplementation administered orally once daily for five consecutive days in the morning. On the fifth day, one hour after the final administration of α-kg, the animals in these groups received castor oil (10 mg/kg, orally) to induce diarrhea. On the same day, the loperamide (LOP) group was pre-treated with LOP, a standard drug, followed by castor oil administration after one hour. The CO group received only castor oil, whereas the vehicle group was treated only with distilled water. Following the administration of castor oil, the animals were placed in boxes lined with absorbent paper and monitored for a period of four hours to assess diarrheal stools. At the end of the protocol, the mice were euthanized using an overdose of ketamine (300 mg/kg) and xylazine (30 mg/kg), which were administered intraperitoneally. The total mass of fecal production (mg) and the total mass of diarrheal feces (mg) excreted by each group were monitored during this period. The efficacy of each treatment is reported as the percentage of diarrhea inhibition (%) compared with the control (CO) group, which was considered to be 100%. The percentage inhibition of defecation and diarrhea was calculated as follows: % Inhibition of diarrhea = [(A - B) / A] × 100, where A represents the mean mass of fecal output induced by castor oil, and B represents the mean mass of fecal output following treatment with the standard drug or α-kg.

### Castor oil-induced enteropooling

Castor oil-induced enteropooling was performed according to the method described by Robert et al. (19), with modifications. The initial experimental procedure followed the steps outlined in the previous section. The efficacy of each treatment is expressed as the percentage of intestinal fluid volume inhibition (%) and calculated as follows: % Reduction of intestinal fluid volume = [(A - B) / A] × 100, where A represents the value of intestinal fluid secretion caused by castor oil, and B represents the value of intestinal fluid in the groups treated with the standard drug or α-kg.

### Evaluation of Cl^-^ ions in intestinal secretion

The assay of chloride ions in intestinal fluid was performed using a colorimetric method with the Cloretos Liquiform kit (Labtest Diagnóstica, Brazil). Samples were centrifuged at a relative centrifugal force of 1,697 *g* at 4°C for 10 min. A 10-µL aliquot of the sample was added to the reagents provided by the standard commercial kit, followed by incubation in a water bath at 37°C for 5 min, and subsequent reading in a spectrophotometer at 480 nm. The concentration of chloride ions was determined using the following formula: [Cl^-^] (mEq/L) = (Abs T) / (Abs P) × 100, where [Cl^-^] represents the concentration of chloride ions in the feces, Abs T is the absorbance of the test sample, and Abs P is the absorbance of the standard.

### Evaluation of NA^+^/K^+^-ATPase activity

The experimental groups were divided as previously described, and diarrhea was induced. For the other protocols, a dose of 120 mg/kg α-kg was used, as it showed the most effective results. Four hours after diarrhea induction, the mice were euthanized and the small intestine (from the pylorus to the cecum) was removed for analysis of Na^+^/K^+^-ATPase activity in enterocytes, according to the method described by Bewaji et al. (20), with modifications. Enzymatic activity was determined based on the protein concentration in the tissue (Labtest Diagnóstica) and Na^+^/K^+^-ATPase activity was calculated by the equation: Specific activity (µmol Pi·mg protein^-1^·h^-1^) = [Pi] × 2 × dilution factor/1000 × protein concentration (mg/mL), where [Pi] represents the concentration of inorganic phosphate in nmoles (according to the calibration curve), 2 represents the factor required to obtain the amount of Pi released per hour, and 1000 represents the factor introduced to convert the Pi released into µmoles.

### Biochemical evaluations

#### Determination of malondialdehyde (MDA) levels in the small intestine

To assess tissue damage following treatment, the methodology was performed according to Mihara et al. (21). MDA concentrations were reported as nmol/g of tissue.

#### Determination of nitrite and nitrate (NOx) levels

NOx levels were measured indirectly through their metabolites, using the Griess reagent, according to the method described by Green et al. (22) and reported as micromoles of NOx.

#### Determination of reduced glutathione (GSH) levels in the small intestine

GSH concentration in intestinal tissues was estimated using the technique described by Sedlak et al. (23) and reported as μg GSH/g wet tissue.

### Histomorphometric analysis

The thickness (μm) of the muscularis, submucosa, and mucosa, as well as the width and depth of the crypts, were assessed under 200× magnification on HE-stained sections. Sixty-four measurements were obtained for each parameter across the entire circumference of the jejunum in at least five animals. Images at 100× magnification were used to measure the height and width (at three points) of 64 enterocytes, as well as the smallest and largest diameters of their nuclei. The height and width of the villi were measured in the jejunum. Villus width was calculated as the average of three measurements taken at the base, middle third, and tip of each villus. The villus-to-crypt ratio was calculated by dividing the height of the villi by the height of the corresponding crypts. One hundred measurements were made for each parameter studied, with 10 measurements per parameter for each mouse. For this analysis, 16 images (4 per quadrant per mouse from 10 mice per group) were acquired with a high-resolution camera, connected to an optical microscope (Nikon Eclipse TS2R, Japan) with 100× magnification and transferred to a microcomputer. Measurements were performed using Image-Pro Plus software (Media Cybernetics, USA) (24).

### Analysis of mucus content in intestinal tissue

Periodic Acid-Schiff (PAS)-stained sections were used to assess the mucus content in the intestinal mucosa after diarrhea induction. For this, 30 images were taken per group, distributed among the animals, captured with a high-resolution camera, connected to an optical microscope (Nikon Eclipse TS2R) with 200× magnification and transferred to a microcomputer. Positive pixels of neutral mucin-like glycoproteins were stained with PAS and quantified using ImageJ® software version 1.53 k (NIH, USA). The results are reported as a percentage of the stained mucin area (1). Measurements were performed using Image-Pro Plus software.

### Statistical analysis

Data are reported as means±SE of 5 animals in each group. Statistical analysis was performed using Graphpad Prism software (version 6.0, USA). The statistical significance of differences between groups was determined by one-way analysis of variance (ANOVA) followed by Tukey's test. Differences were considered significant when P<0.05.

## Results

### Molecular docking

**Figure 1:**
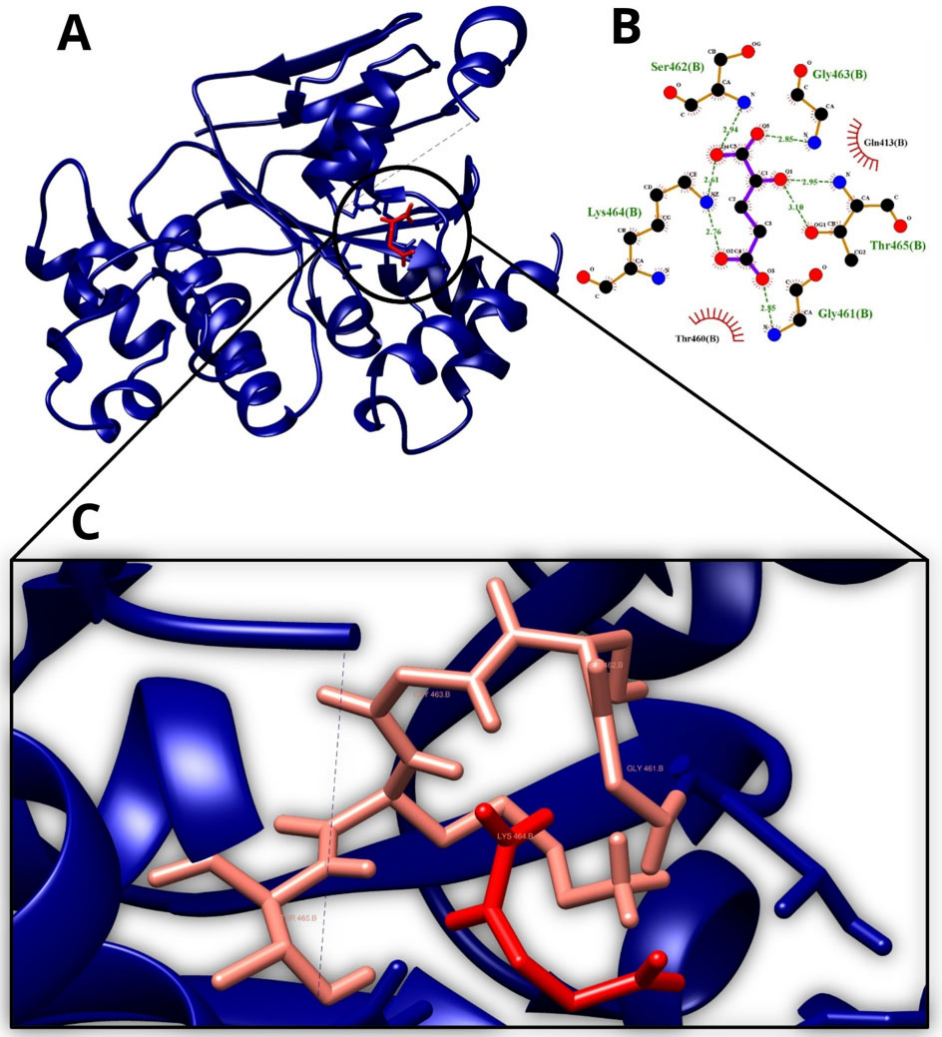
3D molecular docking of the alpha-ketoglutarate (α-kg)/3SI7 complex. **A**, Active binding site between α-kg and 3SI7 (CFTR). **B** and **C**, Respective hydrogen-bonding and hydrophobic interactions.

The optimal molecular affinity parameters were determined by analyzing the interaction between the α-kg ligand and the CFTR protein (PDB ID: 3SI7); the complex exhibited a binding energy of −6.37 kcal/mol and an inhibition constant of 21.31 µM (Table 1, Figure 1). At the active site, the ligand formed hydrogen bonds with the amino acids Ser462, Lys464, Gly461, Thr465, and Gly463, indicating regions where strong molecular interactions occurred. Additionally, two hydrophobic interactions were identified at this site with the amino acids Thr460 and Gln413.

**Table 1:** Molecular affinity parameters of alpha-ketoglutarate (α-kg) with proteins 3SI7, 5OYB, and 3B8E.

Complex (ligand-protein)	ΔG_bind_^a^ (kcal/mol)	Ki^b^	Amino acids that interact through hydrogen bonds^c^	Amino acids that perform hydrophobic interaction^c^
α-kg/3SI7 (CFTR)	-6.37	21.31 µM	Ser462, Lys464, Gly461, Thr465, Gly463	Thr460, Gln413
α-kg/5OYB (CaCC)	-4.59	431.15 µM	-	Asn650, Phe653, Gln649, Ile551, Lys645
α-kg/3B8E (Na^+^/K^+^-ATPase)	-2.88	7.8 µM	Gln699	Asp722, Lys720, Gln274, Gly273, Gly272, Ala721

^a^Binding energy in the best conformation; ^b^Inhibition constant in the best conformation; ^c^Obtained with the Ligplot program.

**Figure 2:**
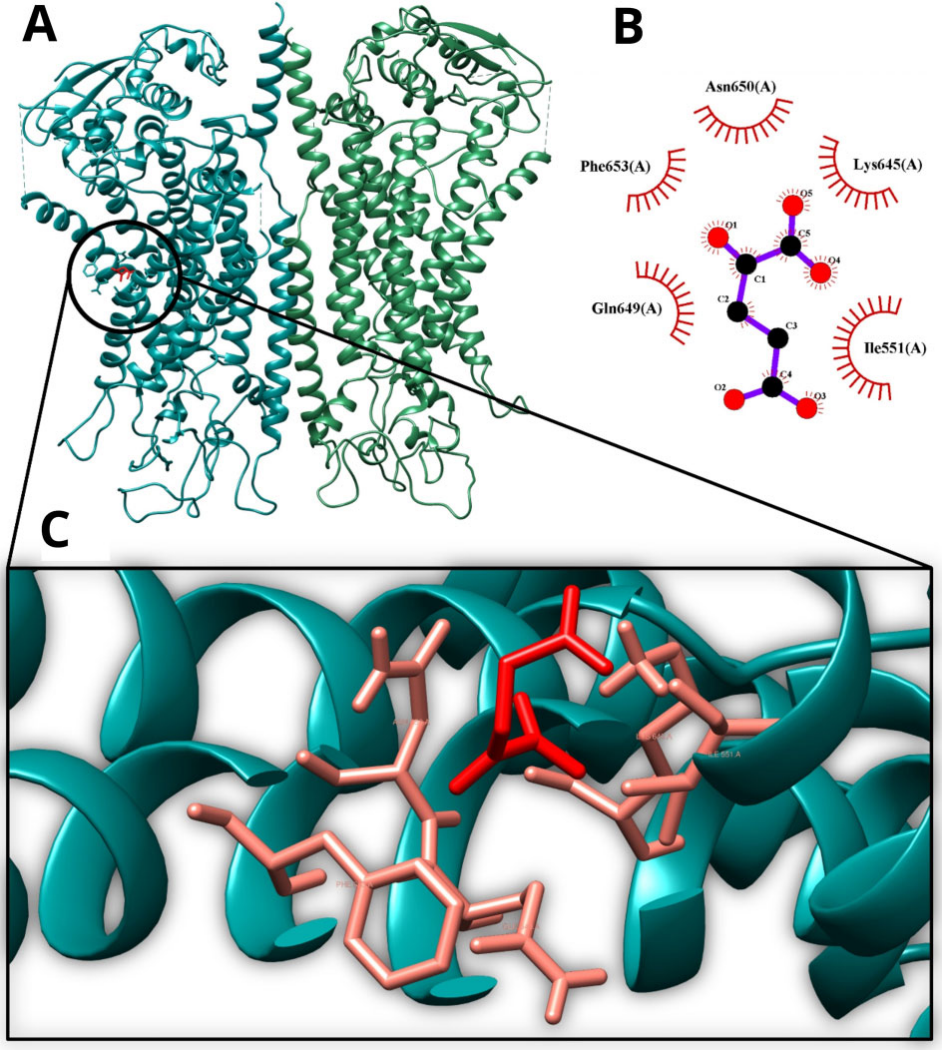
3D molecular docking of the alpha-ketoglutarate (α-kg)/5OYB complex. **A**, Active binding site between α-kg and 5OYB (CaCC). **B** and **C**, Respective hydrophobic interactions.

In addition to its interaction with the aforementioned complex, a favorable molecular affinity was also observed between the α-kg ligand and the protein 5OYB, as shown in Table 1 and Figure 2. This interaction exhibited a binding energy of −4.59 kcal/mol and an inhibition constant of 431.15 µM. This complex did not form any hydrogen bonds; only hydrophobic interactions were observed at the active site, involving the amino acids Asn650, Phe653, Gln649, Ile551, and Lys645 (Table 1).

The interaction between the α-kg ligand and the protein 3B8E also showed significant molecular affinity, with a binding energy of −2.88 kcal/mol and an inhibition constant of 7.8 µM. This complex formed a single hydrogen bond at the active site with the amino acid Gln699. In addition, six hydrophobic interactions were observed at the same site, involving the amino acids Asp722, Lys720, Gln274, Gly273, Gly272, and Ala721 (Table 1).

### Antidiarrheal activity of α-kg

The data obtained in this study demonstrated that castor oil administration induced diarrhea. Supplementation with α-kg at doses of 30, 60 and 120 mg/kg significantly reduced (P<0.05) total fecal mass (1.834 g, 31.77%; 0.660 g, 75.44% and 1.461 g, 45.6%, respectively) compared with the CO group (2.688 g), as well as diarrheal fecal mass (1.636 g, 34.21%; 0.514 g, 79.33% and 0.862 g, 65.33%, respectively) compared with the same group (2.487 g) (Table 2). Among the doses evaluated, the 30 mg/kg dose showed the lowest effect.

**Table 2:** Effect of alpha-ketoglutarate (α-kg) on castor oil (CO)-induced diarrhea in mice.

Treatment	Dose	Total amount of feces (g)	Inhibition of defecation (%)	Total amount of diarrheal stool (g)	Inhibition of diarrhea (%)
Vehicle	2.5 (mL/kg)	0.292±0.000	-	0	-
CO	10 (mL/kg)	2.683±0.003^#^	0.0	2.487±0.000	0.0
α-kg + CO	30 (mg/kg)	1.834±0.001*	31.77	1.636±0.000*	34.21
	60 (mg/kg)	0.660±0.000*	75.44	0.514±0.000*	79.33
	120 (mg/kg)	1.461±0.000*	45.64	0.862±0.000*	65.33
LOP + CO	5 (mg/kg)	0.733±0.000	72.73	0.543±0.000*	78.16

Data are reported as means±SE. Five animals were used per group. *P<0.05 compared with the CO group; ^#^P<0.05 compared with the vehicle group. Results were analyzed by one-way ANOVA followed by Tukey's test. LOP: loperamide (positive control); Vehicle: distilled water.

Enteropooling analysis was conducted to evaluate the volume of intestinal fluid produced during the diarrheal process. Supplementation with α-kg at doses of 30 and 60 mg/kg did not significantly reduce the volume of intestinal content (32.37 and 31.95%, respectively) compared with the CO group. In contrast, α-kg at a dose of 120 mg/kg significantly reduced (P<0.05) intestinal fluid volume by 56.49% compared with the CO group. LOP significantly reduced intestinal content volume by 56.70% compared with the CO group. Our results demonstrated that the 120-mg/kg dose was the most effective and was therefore selected as the standard dose for subsequent experiments (Table 3).

**Table 3:** Effect of alpha-ketoglutarate (α-kg) on castor oil (CO)-induced enteropooling in mice.

Treatment	Dose	Intestinal fluid volume (mL)	Reduction of intestinal fluid volume (mL) (%)
Vehicle	2.5 (mL/kg)	0.100±0.030	-
CO	10 (mL/kg)	0.485±0.045^#^	0.0
α-kg + CO	30 (mg/kg)	0.328±0.049	32.37
	60 (mg/kg)	0.330±0.045	31.95
	120 (mg/kg)	0.211±0.024*	56.49
LOP + CO	5 (mg/kg)	0.210±0.035*	56.70

Data are reported as means±SE. Five animals were used per group. *P<0.05 compared with the CO group; ^#^P<0.05 compared with the vehicle group. Results were analyzed by one-way ANOVA followed by Tukey's test. LOP: loperamide (positive control); Vehicle: distilled water.

#### Effect of α-kg on chloride ion concentration in the intestinal content

The results obtained in this study showed a significant increase (P<0.05) in chloride ion (Cl^-^) concentration in the intestinal content of the CO group (67.87±2.96 mEq/L) compared with the vehicle group (37.84±4.54 mEq/L). Treatment with α-kg significantly reduced (P<0.05) Cl^-^ concentration (29.50±5.45 mEq/L), as did LOP (27.33±3.11 mEq/L) compared with the CO group (Table 4).

**Table 4:** Effect of alpha-ketoglutarate (α-kg) on the concentration of Cl^-^ ions in intestinal content of castor oil (CO)-induced diarrhea in mice.

Treatment	Dose	[Cl^-^] (mEq/L)
Vehicle	2.5 (mL/kg)	37.84±4.54
CO	10 (mL/kg)^#^	67.87±2.96
α-kg + CO	120 (mg/kg)*	29.50±5.45
LOP + CO	5 (mg/kg)*	27.33±3.11

Data are reported as means±SE. Five animals were used per group. *P<0.05 compared with the CO group; ^#^P<0.05 compared with the vehicle group. Results were analyzed by one-way ANOVA followed by Tukey's test. LOP: loperamide (positive control); Vehicle: distilled water.

#### NA^+^/K^+^-ATPase activity in the small intestine

**Figure 3:**
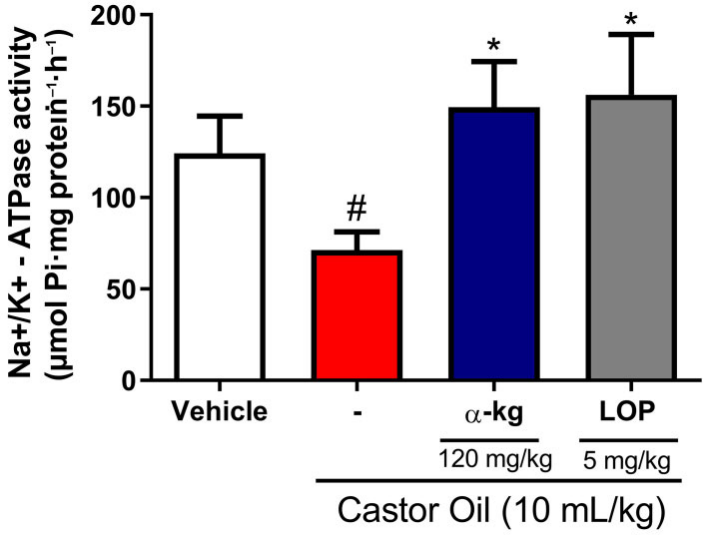
Effect of alpha-ketoglutarate (α-kg) on Na^+^/K^+^-ATPase activity in castor oil-induced diarrhea in mice. Data are reported as means±SE. *P<0.05 compared with the castor oil group; ^#^P<0.05 compared with the vehicle group (distilled water). Data were analyzed by one-way ANOVA followed by Tukey's test; n=5 animals/group. LOP: loperamide (positive control).

The results of this study showed that castor oil induced diarrhea and significantly reduced (P<0.05) Na^+^/K^+^-ATPase activity in the CO group (71.26±5.73 µmol·mg^-1^·h^-1^) compared with the vehicle group (124.30±9.02 µmol·mg^-1^·h^-1^) (Figure 3). However, supplementation with α-kg at a dose of 120 mg/kg significantly increased (P<0.05) Na^+^/K^+^-ATPase activity (149.50±12.47 µmol·mg^-1^·h^-1^) compared with the CO group (Figure 3). Pre-treatment with the standard drug also significantly increased (P<0.05) the Na^+^/K^+^-ATPase activity (156.2±16.53 µmol·mg^-1^·h^-1^). These findings suggest that the restoration of Na^+^/K^+^-ATPase activity may contribute to the antisecretory effect observed in this model, thereby reducing the accumulation of intestinal contents.

#### Effect of α-kg on oxidative stress parameters and reduced glutathione levels

**Figure 4:**
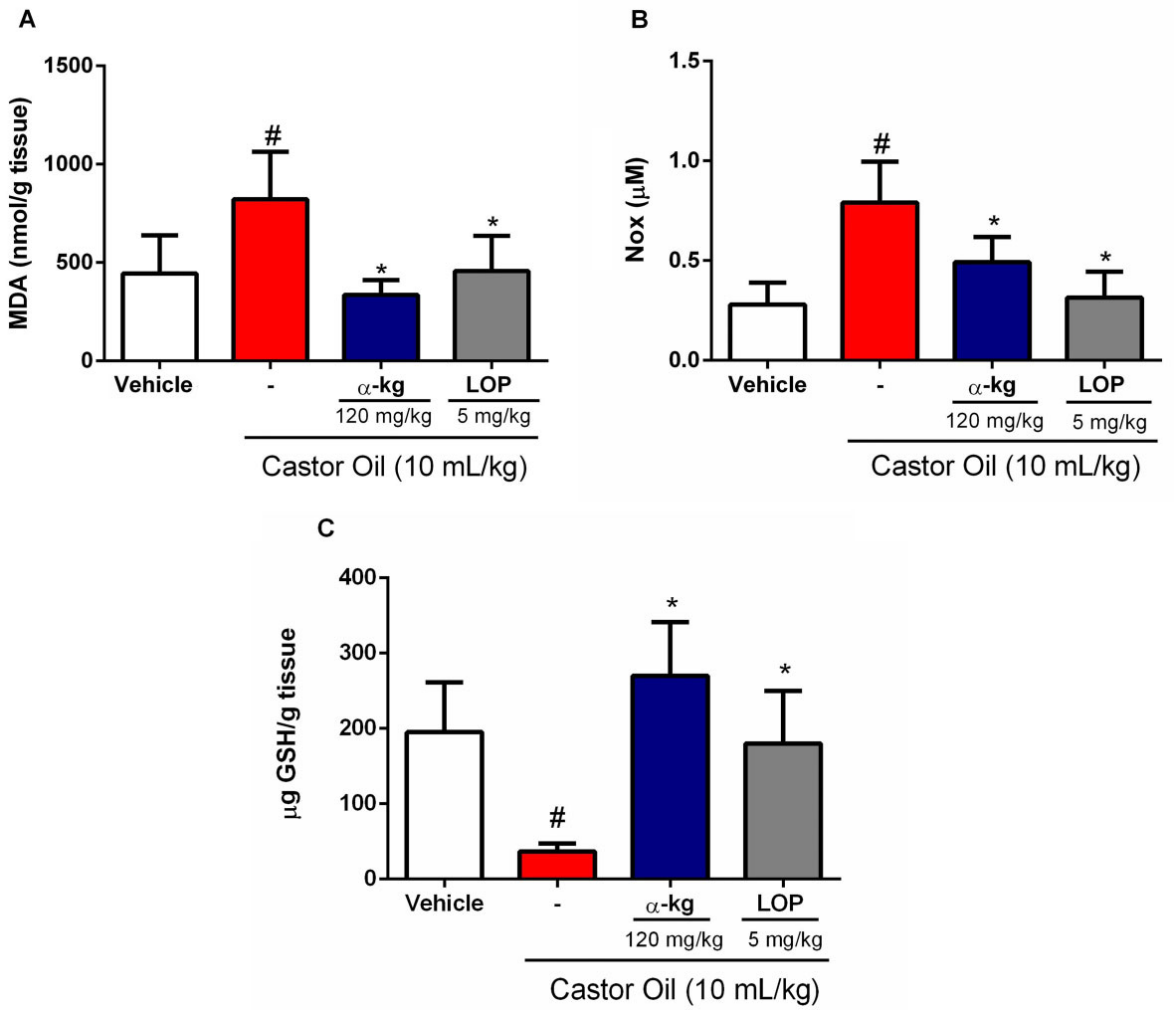
Effect of alpha-ketoglutarate (α-kg) on oxidative and antioxidant stress parameters in castor oil-induced diarrhea in mice. **A**, Castor oil increased malondialdehyde (MDA) levels in the intestine, whereas pre-treatment with α-kg significantly reduced this effect; **B**, Castor oil increased nitrate/nitrite (NOx) levels in the intestine, which were significantly reduced by α-kg pre-treatment; **C**, Castor oil decreased glutathione (GSH) levels, which was restored by α-kg pre-treatment. Data are reported as means±SE. *P<0.05 compared with the castor oil group; ^#^P<0.05 compared with the vehicle group (distilled water). Data were analyzed by one-way ANOVA followed by Tukey's test; n=5 animals/group. LOP: loperamide (positive control).

To evaluate oxidative stress induced by castor oil, biochemical markers were measured. In the evaluation of lipid peroxidation in castor oil-induced diarrhea, a significant increase (P<0.05) in MDA levels (821.50±108.0 nmol/g) was observed in the CO group. Pre-treatment with α-kg significantly reduced (P<0.05) MDA levels (336.10±33.35 nmol/g) (Figure 4A) compared with the CO group, demonstrating its effectiveness in reducing free radicals during diarrheal pathophysiology. Similarly, animals pre-treated with LOP also showed a significant reduction (P<0.05) in MDA levels (456.9±79.97 nmol/g). Castor oil administration significantly increased (P<0.05) NOx levels in the small intestine of the CO group (0.7902±0.083 µM) (Figure 4B) compared with the vehicle group (0.2799±0.049 µM). The groups pre-treated with α-kg and LOP had significantly reduced (P<0.05) NOx levels (0.4917±0.044 µM and 0.3141±0.045 µM, respectively) (Figure 4B) compared with the CO group.

In addition, antioxidant parameters in the intestinal mucosa were evaluated. As expected, castor oil-induced diarrhea significantly decreased (P<0.05) antioxidant content, particularly reduced GSH, in the CO group (36.92±4.24 µg GSH/g tissue) (Figure 4C). Supplementation with α-kg significantly restored (P<0.05) basal GSH levels (270.0±31.97 µg GSH/g tissue) compared with the vehicle group (195.2±29.70 µg GSH/g tissue). Similarly, LOP pre-treatment maintained basal GSH levels (180.1±26.54 µg GSH/g tissue).

### Histomorphometric evaluation

**Figure 5:**
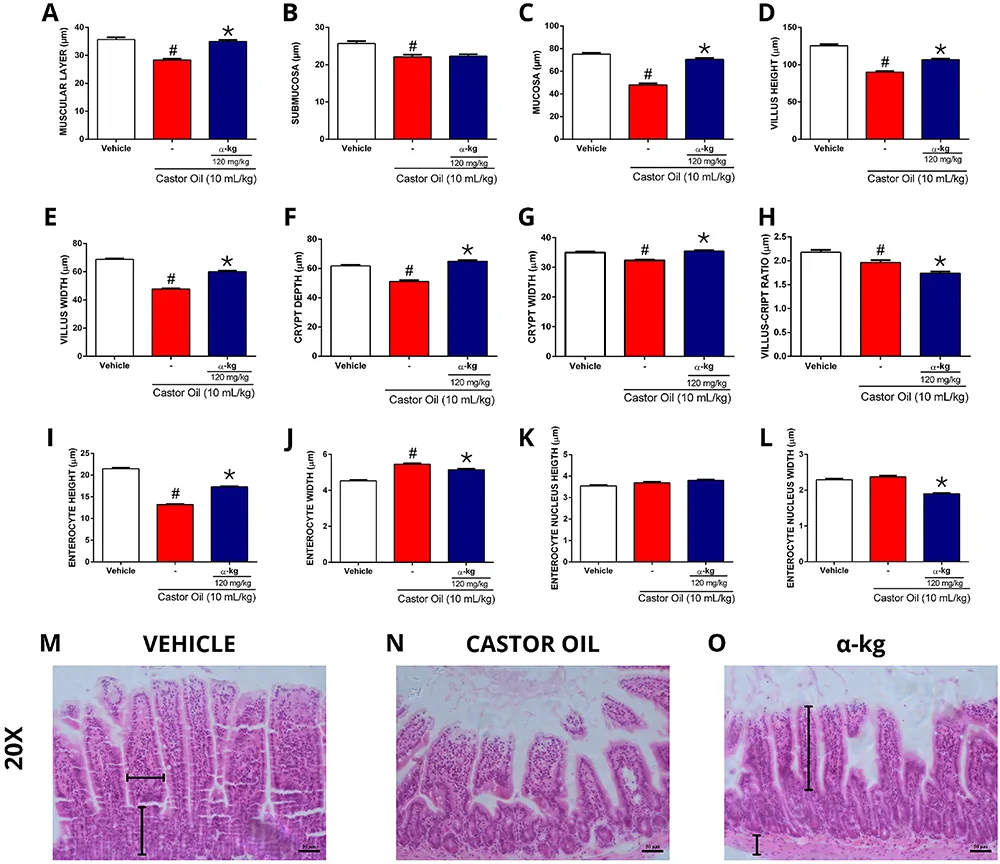
Effect of alpha-ketoglutarate (α-kg) supplementation on duodenal histomorphometry in a murine model of castor oil-induced diarrhea (HE). **A**, Graphical analysis of muscular layer thickness (µm); **B**, submucosa thickness (µm); **C**, mucosa thickness (µm); **D**, villus height (µm); **E**) villus width (µm); **F**, crypt depth (µm); **G**, crypt width (µm); **H**, villus-crypt ratio (µm); **I**, enterocyte height (µm); **J**, enterocyte width (µm); **K**, enterocyte nucleus height (µm); **L**, enterocyte nucleus width (µm). *P<0.05 compared with the castor oil group; ^#^P<0.05 compared with the vehicle group. Data were analyzed by one-way ANOVA followed by Tukey's test (n=5 animals/group). Representative images of HE staining: **M**, vehicle group (negative control; distilled water); **N**, castor oil group (positive control); **O**, α-kg group (120 mg/kg supplemented group). Scale bars 50 μm.

Histomorphometric analysis revealed atrophy of the muscular, submucosal, and mucosal layers in the CO group compared with the vehicle group, whereas these layers were preserved in animals supplemented with α-kg (Figure 5A-C). Regarding the histoarchitecture of villi and crypts, castor oil administration resulted in reduced crypt depth and width, as well as decreased villus height and width, compared with the vehicle group (Figure 5D-G, M, and N). Supplementation with α-kg significantly improved these morphometric parameters compared with the CO group. However, α-kg administration was associated with a significant reduction (P<0.05) in the villus-to-crypt ratio compared with the CO group (Figure 5H and O).

Morphological alterations in enterocytes were also observed following castor oil administration. Enterocyte height was decreased in the CO group compared with the vehicle group, an effect that was reversed by α-kg supplementation (Figure 5I). Furthermore, enterocyte width was increased in the CO group compared with the vehicle group (Figure 5J). With respect to nuclear morphology, α-kg administration significantly reduced nuclear width compared with the CO group, whereas no significant differences were observed in enterocyte nuclear height (Figure 5K and L).

### Effect of α-kg on mucin levels in the intestinal tissue

**Figure 6:**
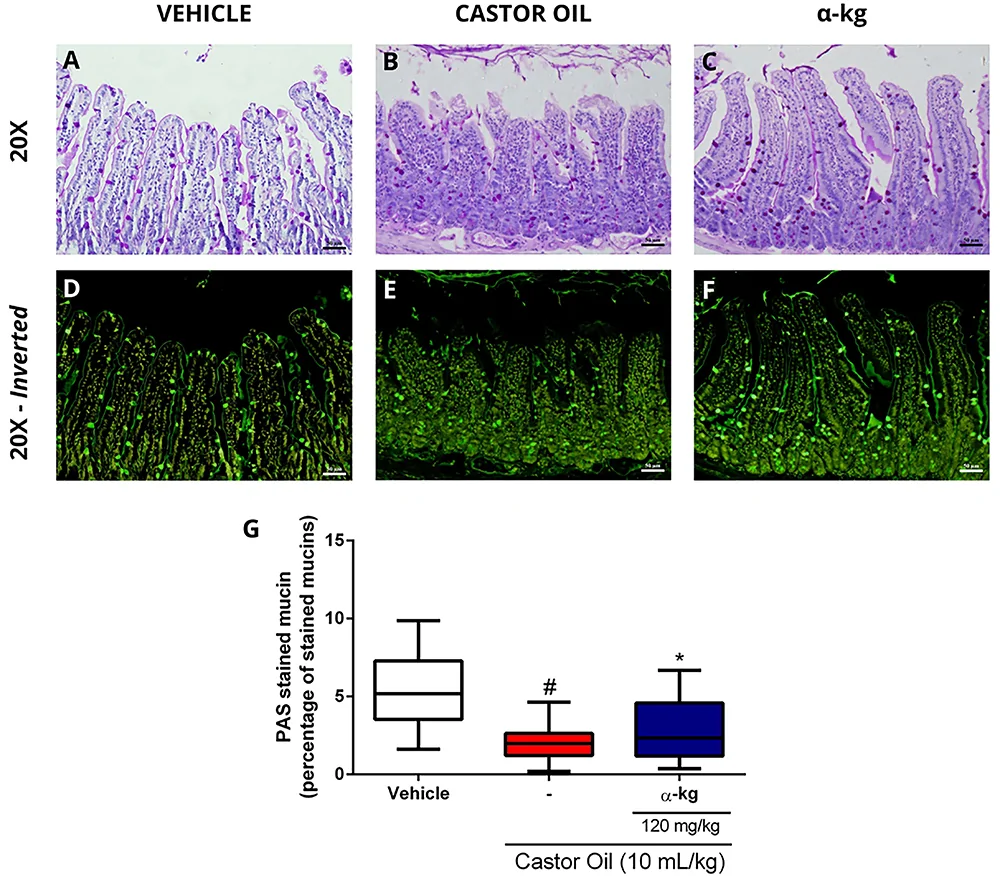
Effect of alpha-ketoglutarate (α-kg) on histological Periodic Acid-Schiff (PAS) staining. The top three photomicrographs are shown at 200× magnification, and the bottom three images are shown at 200× magnification with inverted RGB. Scale bars 50 μm. **A** and **D**, Duodenal samples stained with PAS showing mucin content in the mucosa of the vehicle group (negative control). **B** and **E**, Animals pre-treated with saline followed by castor oil administration (positive control). **C** and **F**, α-kg (120 mg/kg) supplementation group followed by administration of castor oil (supplemented group). **G**, Quantitative analysis of mucin levels based on PAS staining. Data are reported as means±SE. *P<0.05 compared with the castor oil group; ^#^P<0.05 compared with the vehicle group (distilled water). Data were analyzed by one-way ANOVA followed by Tukey's test (n=5 animals/group).

Mucus content was significantly reduced in the CO group (2.022±0.102 pixels/field×10^4^, P<0.05) compared with the vehicle group (5.389±0.218 pixels/field×10^4^). Supplementation with α-kg significantly attenuated diarrhea-induced damage (2.911±0.196 pixels/field×10^4^, P<0.05) and preserved intestinal mucus levels (Figure 6).

## Discussion

Diarrhea, characterized by ionic imbalance, leads to excessive fluid loss and increased gastrointestinal motility (25). Endogenous molecules, such as α-kg, have emerged as promising candidates owing to their antioxidant, anti-inflammatory, and gastroprotective effects (1). Identifying new therapeutic targets remains a complex challenge, often requiring a combination of computational approaches, such as molecular docking, with experimental and clinical studies (26). In this context, we explored the interactions between the α-kg ligand and target proteins involved in the pathophysiology of diarrhea, including CFTR, CaCC, and Na^+^/K^+^-ATPase, using molecular docking as a strategic computational tool.

Among the interactions identified, the binding of α-kg to critical residues of the CFTR channel is particularly noteworthy. This channel consists of five domains: two membrane-spanning domains (MSD1 and MSD2), two nucleotide-binding domains (NBD1 and NBD2), and a regulatory domain (RD). The two MSDs form a pore through which chloride ions (Cl^-^) can pass. These channels can be activated by two distinct mechanisms: i) activation through the NBD1 and NBD2 domains, which contain ATP-binding sites and regulate channel opening via ATP binding and hydrolysis; or ii) phosphorylation of the regulatory domain by protein kinase A (PKA), which is a prerequisite for ATP hydrolysis (27).

Our data demonstrated that α-kg interacted with all analyzed targets, establishing key interactions that contribute to the affinity and stability of the ligand-protein complexes. Among the evaluated targets, the α-kg/CFTR complex exhibited the most favorable interactions. Molecular docking revealed hydrogen bonding between α-kg and specific amino acid residues in the NBD1 region of the CFTR channel, including lysine (Lys464), serine (Ser462), glycine (Gly461-Gly463), and threonine (Thr465), suggesting a potential mechanism underlying modulation of channel activity. These residues are present in critical regions involved in ATP binding and hydrolysis processes essential for the opening and closing of the CFTR channel (28,29).

These amino acids are located close to the Walker A motif of NBD1, a highly conserved region that directly participates in ATP binding. The interaction of α-kg with these residues suggests a potential interference with CFTR's ability to bind ATP, thereby affecting dimerization of NBDs and, consequently, opening of the channel (28).

Our results also demonstrate hydrophobic interactions involving glutamine (Gln413) and threonine (Thr460), which are critical regions associated with the thermal stability of NBD1, channel architecture, NBD1-NBD2 dimerization, ATP hydrolysis, and permeation properties, as evidenced by structural modelling and computational studies (29). Thus, our findings suggest that α-kg may negatively modulate CFTR channel activity, leading to reduced chloride ion secretion in the intestine. This effect could be beneficial in the treatment of conditions such as secretory diarrhea that involves CFTR hyperactivity contributing to excessive fluid loss into the intestinal lumen (30).

Another protein involved in diarrheal processes evaluated in this study was CaCC, a calcium-activated chloride channel, which plays a key role in epithelial secretion and muscle contraction. Its structure comprises a transmembrane (TM) domain and a Ca^2+^-binding site located near the channel opening region (31). Molecular docking analysis revealed hydrophobic interactions with asparagine (Asn650), phenylalanine (Phe653), glutamine (Gln649), isoleucine (Ile551), and lysine (Lys645) residues, which are located in the intracellular domain, close to the Ca^2+^-binding sites, and are potential regions of allosteric modulation. Molecular dynamics studies indicated that interactions with lysine residues may favor formation of electrostatic bonds with ATP phosphate groups, which can directly influence channel conductance. The findings suggest that residues such as Asn650 and Lys645 may participate in the functional modulation of CaCC, possibly by affecting channel gating in response to intracellular calcium (32).

Furthermore, the residues Phe653 and Ile551, identified as being involved in hydrophobic interactions, are located in critical structural regions, particularly in the transmembrane helices that form the channel pore. Evidence from the literature indicates that Phe653 may be positioned in an area that regulates ionic permeability, and therefore, function as a determinant of channel selectivity. Ile551 is associated with a domain that undergoes conformational rearrangements during Ca^2^-dependent activation and is essential for the opening and closing of the channel (33). These findings reinforce the hypothesis that the observed molecular interactions may directly impact CaCC activity, representing a potential therapeutic target in conditions associated with intestinal hypersecretion.

Our computational study further evaluated the interactions of α-kg with Na^+^/K^+^-ATPase (3B8E), revealing significant predicted hydrogen bonding with Gln699 in the P domain, along with hydrophobic interactions involving Asp722, Lys720, Gln274, Gly273, Gly272, and Ala721 across the P and N domains. Previous studies have highlighted that the interactions with the Gln699 residue are associated with the stabilization of the phosphate group during the phosphorylation reaction, a key step in the pumping cycle. Additionally, the critical regions Gly272 and Gly273 are predicted to contribute to the flexibility required for conformational changes during the pumping cycle (34). Similar interaction patterns were observed in our study, along with additional key interactions involving the channel. These findings highlight the relevance of examining how fluctuations in cellular α-kg levels, such as those occurring under diarrheal conditions, may directly influence Na^+^/K^+^-ATPase activity and, consequently, electrophysiological homeostasis. These interactions suggest a multifactorial mechanism by which α-kg may modulate fluid secretion and absorption, supporting its potential as an antidiarrheal candidate.

Castor oil-induced diarrhea has been well described in the literature. Its active component, ricinoleic acid, acts through multiple mechanisms, including the release of nitric oxide and prostaglandins, as well as the inhibition of Na^+^/K^+^-ATPase activity. These actions result in reduced absorption and increased secretion of electrolytes and water, leading to accelerated intestinal transit and enhanced contractility of intestinal smooth muscles (35,36).

Among the parameters used to assess diarrhea, quantitative analysis of total and diarrheal feces and calculation of the percentage inhibition of diarrhea are essential for evaluating the efficacy of the compound under investigation (37). In the present study, α-kg supplementation reduced the total and diarrheal fecal outputs compared with the CO group. These findings are consistent with previous studies demonstrating reduction in defecation following glutamine administration in rats with castor oil-induced diarrhea (36).

Another critical factor in the diarrheal process is the accumulation of intestinal secretion, which was analyzed using the enteropooling assay. Accordingly, our results demonstrated that α-kg supplementation had a significant antisecretory effect, representing the first study to evaluate this specific parameter related to antidiarrheal activity. The observed reduction in intestinal fluid may be related to antisecretory mechanisms involving modulation of the water and electrolyte (Cl^-^) transport across the mucosa and enhanced Na^+^/K^+^-ATPase activity (38). Further, our *in vivo* results are in line with the predictions from computational analysis of key residues in the NBD1 portion of the CFTR channel, suggesting a possible functional inhibition of Cl^-^ secretion and positive regulation of Na^+^/K^+^-ATPase activity.

In the intestinal epithelium, redox imbalance constitutes a central pathophysiological factor contributing to both mucosal barrier dysfunction and development of various gastrointestinal disorders. Evidence indicates that castor oil induces a pro-oxidant state in the intestinal mucosa, characterized by increased production of reactive oxygen species (ROS), lipid peroxidation of cell membranes, and significant depletion of antioxidant defenses (38). Experimental data reveal that α-kg supplementation leads to a decrease in the oxidative biomarkers Nox and MDA, along with the preservation of GSH activity. These findings are consistent with previous reports demonstrating the antioxidant potential of α-kg in the gastrointestinal tract through reduction of mitochondrial ROS production, upregulation of endogenous antioxidant systems, and maintenance of oxidative homeostasis (39).

Previous studies have shown that castor oil induces changes in the histoarchitecture of the intestinal epithelium, characterized by tissue layer atrophy, reduced villus-to-crypt ratio, epithelial thinning, and loss of enterocyte polarity. Ricinoleic acid further disrupts intercellular junction complexes, increasing paracellular permeability and compromising barrier function (35). In our study, we observed impaired epithelial organization in the CO group, whereas intestinal architecture was preserved in animals supplemented with α-kg. In this context, α-kg supplementation has previously been shown to exert significant cytoprotective effects in experimental models of intestinal injury, primarily by promoting epithelial cell proliferation, preserving tight junction integrity, and maintaining intestinal architecture and permeability (40).

In this study, the PAS histochemical technique was employed to evaluate the effects of α-kg on intestinal mucin composition. The mucus layer, which constitutes the first line of defense of the intestinal barrier, is structurally and functionally compromized during episodes of osmotic diarrhea, such as castor oil-induced diarrhea (38). Our results demonstrated that α-kg supplementation maintained the integrity of the mucus layer compared with the CO group, highlighting its cytoprotective role. It has been proposed that α-kg may positively modulate transcription factors involved in mucus production, thus promoting the differentiation of secretory epithelial cells, increasing the proportion of goblet cells, and enhancing mucin synthesis (1).

The outcomes of α-kg supplementation, particularly the attenuation of diarrheal manifestations, provide evidence for the physiological relevance of this endogenous metabolite in maintaining intestinal integrity. These results suggest that α-kg supplementation may represent a promising nutritional strategy to support intestinal health under clinical conditions. However, additional studies are warranted to validate its effectiveness across different populations and to elucidate the molecular mechanisms underlying its protective effects.

## Data Availability

All data generated or analyzed during this study are included in this published article.
